# The Multimorbidity and Lifestyle Correlates in Chinese Population Residing in Macau: Findings from a Community-Based Needs Assessment Study

**DOI:** 10.3390/healthcare11131906

**Published:** 2023-06-30

**Authors:** Qingling Yang, Quanzhi Zhang, Fei Wan Ngai, Shaoling Wang, Dexing Zhang, Yang Gao, Chun Hao, Harry Haoxiang Wang, Oi Ching Bernice Lam Nogueira, Ming Liu, Alex Molasiotis, Alice Loke, Yaojie Xie

**Affiliations:** 1 School of Nursing, The Hong Kong Polytechnic University, Hong Kong SAR, China; 2 School of Nursing, Harbin Medical University, Harbin 150088, China; 3 The Jockey Club School of Public Health and Primary Care, The Chinese University of Hong Kong, Hong Kong SAR, China; 4 Department of Sport, Physical Education, and Health, Hong Kong Baptist University, Hong Kong SAR, China; 5 School of Public Health, Sun Yat-sen University, Guangzhou 510080, China; 6 College of Medicine and Veterinary Medicine, The University of Edinburgh, Edinburgh EH8 9AG, UK; 7 School of Health Sciences and Sports, Macao Polytechnic Institute, Macao 999078, China; 8 Health and Social Care Research Centre, University of Derby, Derby DE22 1GB, UK

**Keywords:** multimorbidity, lifestyle, drinking, BMI, sleeping

## Abstract

Multimorbidity has become one of the most pressing public health concerns worldwide. The objectives of this study were to understand the prevalence of multimorbidity and its relationship with lifestyle factors among Chinese adults in Macau, and to investigate the combined contribution of common lifestyle factors in predicting multimorbidity. Data were collected through face-to-face interviews using a self-reported questionnaire on common chronic diseases, lifestyle factors and sociodemographics. BMI, physical activity, drinking status, smoking status and sleep quality were assessed, and a composite lifestyle score (0 to 9 points) was calculated, and the higher the score, the healthier the lifestyle. A total of 1443 participants were included in the analysis, of whom 55.2% were female, 51.8% were middle aged or elderly and 30.5% completed tertiary education or higher. The prevalence of multimorbidity was 10.3%. The combination of hypertension and hyperlipidaemia was the most common (22.2%) multimorbidity among the participants with multimorbidity. After the adjustment of the covariates, it was found that the participants who were overweight (OR: 1.95, 95% CI: 1.18–3.20, *p* = 0.009) or obese (OR: 3.76, 95% CI: 2.38–5.96, *p* < 0.001), former drinkers (OR: 2.43, 95% CI: 1.26–4.69, *p* = 0.008), and those who reported poor sleep quality (OR: 2.25, 95% CI: 1.49–3.40, *p* < 0.001) had a high risk of developing multimorbidity. A one-unit increase in the lifestyle score was associated with a 0.33-times reduction in the risk of developing multimorbidity (OR: 0.67; 95% CI: 0.59–0.77, *p* < 0.001). A combination of lifestyle factors can influence a variety of multimorbidity among the Chinese adults in Macau. Thus, comprehensively assessing the combined contribution of several lifestyle factors in predicting multimorbidity is important.

## 1. Introduction

Multimorbidity is defined as the coexistence of two or more chronic diseases in a single individual [1]. The prevalence of multimorbidity has grown worldwide in recent years, which has led to heavy disease burdens and high health costs globally [2]. In the United States, the prevalence of multimorbidity among the young population (aged 18–44 years) ranges from 37.9% in the District of Columbia to 64.4% in West Virginia [3]. A population-based study in Southern Brazil reported an overall multimorbidity prevalence of around 30% in 2927 adults [4]. In Hong Kong, a population-based study showed that the prevalence of multimorbidity was 11.1% across all age groups [5]. Meanwhile, another Hong Kong study found that 13.5% of people aged 15 years or above have multimorbidity [6]. The aforementioned epidemiological studies demonstrated a variety of multimorbidity in different countries and regions and that its high prevalence in developed and developing countries can exert a considerable burden on their healthcare system [4,7]. People with multimorbidity are more likely to suffer poorer health outcomes, such as increased mortality [3,8] and disability [9,10], decreased physical function [9,10], reduced quality of life [9,11,12,13], and poorer self-rated health [14,15]. Individuals with multimorbidity also have higher healthcare utilisation, such as the use of primary care services and hospitalisation, which may increase health expenditures [2,8,14].

Many lifestyle factors are associated with multimorbidity. Unhealthy lifestyles, such as physical inactivity [16,17], smoking [5,18,19], drinking [13] and sleep disorders [20] were found to be risk factors for certain chronic diseases and the development of multimorbidity. A significant association was also found between body mass index (BMI) and multimorbidity [21,22,23]. Specifically, people who are overweight or obese are likely to have multimorbidity [24]. Although considerable evidence showed the negative impact of unhealthy lifestyle behaviours on multimorbidity, some studies presented inconsistent results and contrary findings. For example, a study in Canada showed that daily or weekly alcohol consumption can reduce middle-aged people’s odds of developing multimorbidity [25]. Furthermore, most studies investigated only the relationship between a single lifestyle factor and multimorbidity, but individuals’ lifestyle behaviours are complex and volatile. Lifestyle factors can jointly influence individuals’ health status. Thus, comprehensively assessing the combined contribution of several lifestyle factors in predicting multimorbidity is important.

Macau, which is located south of the Pearl River Delta, is a special administrative region of China that is known as the ‘Las Vegas of the East’ because it is home to some of the largest and most popular casinos in the commercial gambling industry in the world [26]. Macau is an international city where Chinese and Western cultures coexist; thus, heavy drinking is common among Macau residents [27]. However, its ageing society places a heavy health burden on Macau [28]. Unhealthy lifestyles are common in Macau, but what is the multimorbidity situation of its residents, and how does it relate to their lifestyle? No relevant studies have focused on this population. Therefore, assessing the multimorbidity and lifestyles of Macau residents can give us a satisfactory profile and an improved understanding of lifestyles and enable us to develop strategies to promote the health of this population. The objectives of this study were to understand the characteristics of multimorbidity in a representative sample of the Chinese population of Macau, determine its association with common lifestyles and evaluate the combined contribution of lifestyle factors in predicting multimorbidity.

## 2. Materials and Methods

### 2.1. Study Design and Settings

Our study was part of the ‘Healthy Life, Longer Lives’ (HLLL) project, which conducted a community-based cross-sectional survey on a representative sample of community-dwelling people in Macau, from 2017 to 2019. The HLLL project was designed to investigate the lifestyle behaviours of Macau residents in terms of their dietary habits, sleep patterns, physical activity (PA), electronic device usage, smoking, drinking and health information seeking, and to identify the barriers to a healthy lifestyle in vulnerable groups. We obtained data from adults (aged ≥ 18 years) to identify the multimorbidity situation and related lifestyle factors in the population. 

### 2.2. Ethical Considerations

This project was approved by the institutional review boards of The Hong Kong Polytechnic University (HSEARS20180516001), the Kiang Wu Nursing College of Macau (REC-2017.3) and the Macau Polytechnic Institution (07/PSC/ESS/2017). All the participants provided their written informed consent.

### 2.3. Participants

Our target population was adults of Chinese descent residing in Macau. The inclusion criteria included individuals who (1) were 18 years old or older, (2) could read and write Chinese and speak Cantonese or Putonghua and (3) were currently residing in one of the seven parishes of Macau. The exclusion criteria were (1) individuals of mixed descent, that is, Chinese and other ethnicities; (2) nonpermanent residents; (3) individuals who were unable to communicate owing to psychological or physical illnesses; and (4) individuals who were unable to provide written informed consent.

We invited a total of 1623 Macau residents to participate in the study, 122 of whom declined our invitation. Of the 1501 individuals who agreed to participate in the study, 57 were below the age of 18 years, and 1 refused to provide written informed consent. Ultimately, a total of 1443 individuals completed the questionnaire and were included in the analysis (Figure S1).

### 2.4. Sampling and Recruitment

We estimated the sample size using the formula $n=Z_{\alpha /2}^{2}\times P(1-P)$/$d^{2}$, where $Z_{\alpha /2}$ is the standard normal deviate corresponding to the desired confidence level, P is the estimated prevalence of the outcome and d is the desired margin of error. Assuming that the expected prevalence of multimorbidity was 1.3% [5], and setting the accepted error as 0.02 at the 95% confidence level, we expected to obtain 1087 successful responses. With reference to the age and gender distributions in the Macau Census data [29], we determined the number of required participants in each age group (18–24, 25–34, 35–44, 45–54, 55–64 and 65+ years; Table S1). We trained 30 baccalaureate nursing students from two tertiary education institutions in Macau to complete recruitment and data collection. The students lived in different parishes in Macau and recruited the participants from their district of residence as well as from nearby housing districts. We invited only one eligible person per household to participate in the study to ensure the representativeness of the sample. In addition, to identify the participants, we asked them to provide the last three digits of their Macau national identification number. After the participants gave their written informed consent, we gave them the self-administered questionnaire to collect their information. For the participants who could not read the questionnaire, such as the elderly, our student assistants conducted a face-to-face interview to collect their information. According to the feedback provided by the student assistants, only a small proportion of the elderly participants (aged ≥ 75 years), that is, less than 1% of the whole sample, completed the questionnaire through an interview.

### 2.5. Measures

#### 2.5.1. Assessment of Chronic Diseases

By referencing the classification of chronic diseases of previous studies [12,30,31], we evaluated the 11 most common physical chronic diseases in the target population, namely, osteoporosis, hyperlipidaemia, hypertension, diabetes, chronic obstructive pulmonary disease, hepatitis, heart disease, asthma, stroke, cancer and migraine. We instructed the participants to self-report any doctor-diagnosed chronic diseases on the questionnaire, which the student assistants verified with the participants to ensure accuracy.

#### 2.5.2. Identification of Multimorbidity

Multimorbidity was defined as the coexistence of two or more chronic diseases in a single person [31,32]. We categorised the participants as having multimorbidity or not having multimorbidity (without a chronic disease or with one chronic disease) for the analysis.

#### 2.5.3. Lifestyle Factors and Anthropometrics

*PA*. We measured PA using the International Physical Activity Questionnaire-Short Form (IPAQ-SF) [33], which is a self-administered seven-item questionnaire designed to measure an individual’s PA over the past seven days, including work, transportation and recreational activities. We calculated the total metabolic equivalent according to the number of minutes per day or per week that the individual engaged in each activity. We asked the participants to recall the duration and frequency of their vigorous PA (VPA) and moderate PA (MPA). Then, we calculated the total PA (TOPA) by summing their VPA and MPA. We classified PA into two levels, according to the recommendations of the World Health Organization (WHO) [34]: enough (≥150 min of MPA/week or ≥75 min of VPA/week) and not enough (<150 min of MPA/week or <75 min of VPA/week). The intraclass correlation coefficient of the Chinese version of the IPAQ was 0.79 [35].

*Drinking*. We evaluated their drinking status by asking the participants whether they had a habit of drinking alcohol. We grouped the participants into three categories according to their drinking status: current drinker, former drinker who has quit drinking and has never drunk alcohol. If a participant was a current drinker, then we asked their drinking frequency, consumption amount and preferred type of drink [32]. We assessed the drinking status by calculating the participants’ total number of alcoholic drinks (1 drink was equivalent to 10 g of ethanol) consumed in a week [34]. 

*Smoking*. We collected information on the participants’ smoking status and classified them as nonsmokers, former smokers or current smokers [34]. In addition, we asked the participants their preferred cigarette type, such as e-cigarettes, cigarettes, self-rolling cigarettes or cigars. Then, we asked the participants who we identified as smokers how many cigarettes they smoked per day and whether they had tried to quit in the past year [32].

*Sleep quality*. We used the Chinese version of the Pittsburgh Sleep Quality Index (PSQI) to measure sleep quality [36]. The PSQI uses 19 items to assess subjective sleep quality in the past month. The items are divided into seven components: sleep duration, sleep disturbance, sleep latency, daytime dysfunction owing to sleepiness, sleep efficiency, overall sleep quality and sleep medication. Each item was scored from 0 to 3. We added the scores of the seven components to generate an overall score that ranged from 0 to 21, and the higher the score, the worse the sleep quality. A global PSQI score of > 5 indicates poor sleep or insomnia. The Chinese version of the PSQI demonstrated satisfactory reliability (r = 0.82–0.83) and a sensitivity of 98% [36].

*BMI*. We asked the participants to report their current weight and height measured by standard scales. We recorded their height to the nearest 0.1 cm and weight to the nearest 0.1 kg. The BMI is calculated as weight in kilograms (kg) divided by height in meters (m) squared BMI (kg/m^2^). We classified BMI into four levels according to the WHO standards for Asian populations [37]: underweight (<18.5 kg/m^2^), normal weight (18.5–22.9 kg/m^2^), overweight (23.0–24.9 kg/m^2^) and obese (≥25.0 kg/m^2^). We further grouped the four categories as underweight/normal weight (<23.0 kg/m^2^), overweight (23–24.9 kg/m^2^) and obese (≥25 kg/m^2^) for the analysis.

#### 2.5.4. Lifestyle Score Calculation

We calculated the total lifestyle score using an algorithm that summarised the subscores of BMI, TOPA, drinking, smoking and sleep quality [34]. We categorised the first four lifestyle factors into three levels: 0 (poor), 1 point (intermediate) and 2 points (optimal). We used the lifestyle score calculation method from our previous study [32], as shown in Table 1. For BMI, the three levels were <23 kg/m^2^, 23–24.9 kg/m^2^ and ≥25 kg/m^2^. With regard to TOPA, 0 min was considered as poor, and less than 150 min and 150 min or more were considered as intermediate and optimal, respectively. For drinking, ≥ 8 drinks/week for the women and ≥ 15 drinks/week for the men were considered as poor, and no drinking and 1–7 drinks/week for the women and 1–14 drinks/week for the men were considered as intermediate and optimal, respectively [38,39]. For smoking, the three levels were current smokers (poor), former smokers (intermediate) and nonsmokers (optimal). We categorised sleep quality into two levels: a PSQI score of >5 (0 point) and a PSQI score of ≤5 (1 point), and the higher the overall score, the healthier the overall lifestyle (Table 1).

#### 2.5.5. Socio-Demographics

We obtained the participants’ sociodemographic information, including their gender, age, education level, marital status, employment status and monthly family income. We recorded age as a continuous variable (years) and categorised it into three groups: 18–44 years, 45–64 years and ≥65 years. We defined education level as primary or lower, secondary and tertiary or higher. We dichotomised marital status as single (i.e., never married, separated, divorced or widowed) or coupled (i.e., married or living with a partner). We classified employment status as employed, unemployed and other (i.e., retired, housewife, unable to work or student). Finally, we grouped monthly family income into four categories (MOP): 29,999 or less, 30,000–59,999, 60,000 or more and unknown (USD 1 = MOP 8.03).

### 2.6. Statistical Analysis

We performed the descriptive analysis using the mean and standard deviation (SD), frequency and percentage for the continuous variables and categorical variables. We used the chi-squared test for the categorical variables and independent *t*-test for the continuous variables to compare the variable differences between the different multimorbidity statuses.

We employed logistic regression models to examine the associations between the lifestyle factors and lifestyle score and multimorbidity of the Macau population. Model 1 was the univariate logistic regression model, with only one lifestyle factor or lifestyle score as the independent variable, and Model 2a was the multivariable logistic regression model. We included BMI, VPA, MPA, drinking status, smoking status and insomnia in one model for the analysis and made adjustments for gender, age, employment status, education level, marital status and monthly family income. The adjustment variables in Model 2b were the same as those in Model 2a, and we included the lifestyle score in the model for the analysis. Additionally, we performed priori stratification analyses by gender and age (stratified by 45 years) to assess the potential moderating effect of such factors on the association between the lifestyle characteristics and multimorbidity.

We conducted several sensitivity analyses to test the robustness of our findings by (1) excluding the participants with only one chronic disease from the analysis, (2) excluding the participants with no chronic disease from the analysis and (3) constructing a multivariable logistic regression model that included BMI, TOPA, drinking status, smoking status, insomnia and the other covariates for the analysis.

We conducted all the statistical analyses using R 4.2.1 (R Foundation for Statistical Computing). A two-sided *p* < 0.05 was considered to be statistically significant.

## 3. Results

### 3.1. Basic and Lifestyle Characteristics of the Participants

We summarised the basic and lifestyle characteristics of the participants based on their multimorbidity status in Table 2. Among the participants, 55.2% were women, 51.8% were middle aged or elderly, 30.5% completed tertiary education or higher and around half (49.8%) had a monthly family income of less than MOP 29,999 (USD 1 = MOP 8.03). Compared with the participants with no multimorbidity, the participants with multimorbidity were older, completed primary education or lower, were coupled and were retired/a housewife (all *p* < 0.05).

In terms of their lifestyle characteristics, around one-fifth of the participants were obese (19.9%) and had enough VPA (19.8%) and MPA (20.9%), 31.5% had enough TOPA, 25.3% were current drinkers, 8.0% were current smokers and 32.3% reported poor sleep quality. The mean lifestyle score was 6.09. 

### 3.2. Prevalence of Chronic Diseases and Multimorbidity in the Study Population

**Figure 1:**
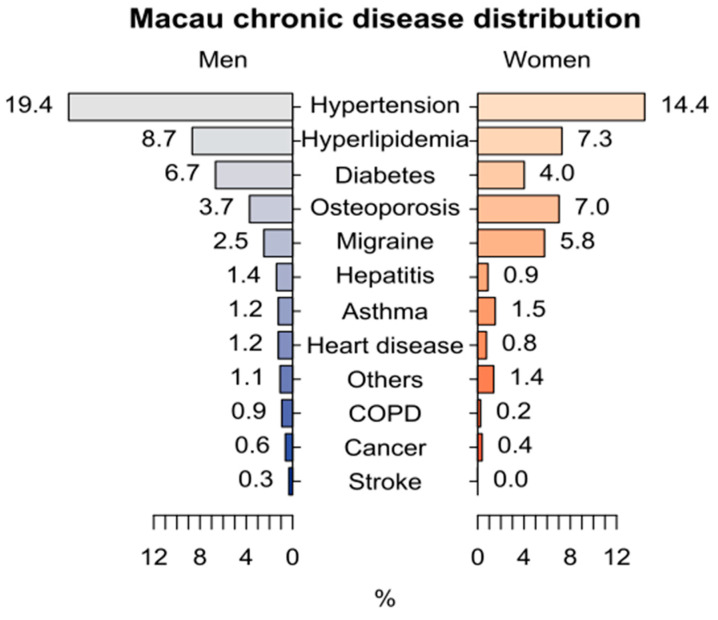
Chronic disease distribution of Macau population.

The distribution of single chronic diseases according to gender are shown in Figure 1. Hypertension and hyperlipidaemia were the two most common chronic diseases in the male and female participants (19.4% and 8.7%, respectively, for the male participants and 14.4% and 7.3%, respectively, for the female participants). Diabetes was the third most common chronic condition in the male participants (6.7%), whereas osteoporosis was the third most common chronic disease in the female participants (7.0%). Migraine was 2.3 times more common in the female participants than in the male participants (5.8% vs. 2.5%). 

**Figure 2:**
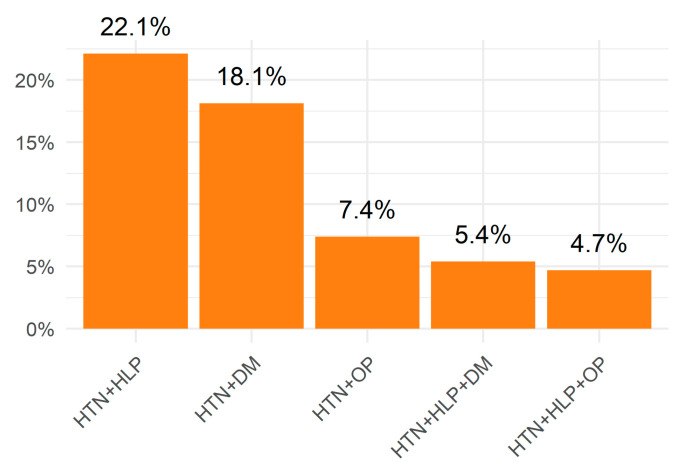
Top five disease combinations among Macau population with multimorbidity. HTN, hypertension; HLP, hyperlipidaemia; DM, Diabetes mellitus; OP, osteoporosis.

The prevalence of multimorbidity was 10.3%. Among the participants with multimorbidity, 71.1% experienced the co-occurrence of two conditions, 21.5% had three conditions, 5.4% had four conditions and only 2.0% had five or more conditions. In addition, among the participants with multimorbidity, the most common disease combination was the co-occurrence of hypertension and hyperlipidaemia (22.2%), followed by the co-occurrence of hypertension and diabetes (18.1%); hypertension and osteoporosis (7.4%); hypertension, hyperlipidaemia and diabetes (5.4%); and hypertension, hyperlipidaemia and osteoporosis (4.7%; Figure 2).

### 3.3. Association between Lifestyle Factors and Lifestyle Score and Multimorbidity

In the univariate analysis, BMI, VPA, drinking, smoking, sleep quality and the lifestyle score were positively significantly associated with multimorbidity (all *p* < 0.05; Table 3). After adjusting for the other lifestyle factors and covariates, including gender, age, employment status, education level, marital status and monthly family income, with reference to the underweight/normal weight participants, we determined that the participants who were obese were 3.76 (95% CI: 2.38–5.96, *p* < 0.001) times more likely to have multimorbidity (Table 3). The participants who had quit drinking had 2.43 (95% CI: 1.26–4.69, *p* = 0.008) times higher odds of having multimorbidity than those who had never drunk alcohol, whereas no significant difference existed between the participants who were drinkers and those who have never drunk alcohol (*p* = 0.24; Table 3). Regarding sleep quality, the participants who suffered from insomnia had 2.25 (95% CI: 1.49–.40, *p* < 0.001) times higher odds of having multimorbidity than those who did not suffer from insomnia (Table 3). For the overall lifestyle score, after adjusting the covariates, we observed that a one-unit increase in the score decreased the odds of having multimorbidity by 36% (OR: 0.64; 95% CI: 0.57–0.72, *p* < 0.001; Table 3).

In the stratification analysis by gender, we found a significant association between BMI, insomnia, the lifestyle score and multimorbidity in the men and women, but drinking status was associated with multimorbidity only in the men and not in the women (Table S2). The results of the stratification analysis for the participants aged 45 years or older were consistent with the main results, but for the participants younger than 45 years, only BMI and the lifestyle score were significantly associated with multimorbidity (Table S3).

The exclusion of the participants with only one chronic disease or no chronic disease did not qualitatively alter the association between BMI, drinking status, insomnia and the lifestyle score and multimorbidity (Tables S4 and S5). Furthermore, the inclusion of TOPA in the multivariable model did not substantially alter the estimates (Table S6). 

## 4. Discussion

To the best of our knowledge, our study is the first to use a comprehensive and population-based approach to estimate the prevalence of multimorbidity in a large sample of the Macau population. The findings can help raise awareness of the burden of multimorbidity and the importance of addressing its risk factors. In addition to a few common lifestyle factors, such as BMI, drinking and sleep quality, we found that the overall lifestyle score can quantify the combined impact of the lifestyle factors on the health of the participants from Macau. Our findings can serve as a basis for the development and implementation of effective interventions to prevent and manage multimorbidity and thus alleviate people’s suffering and social burden.

Overweight and obese people have a higher risk of having multimorbidity than those with normal weight. The number of overweight individuals has increased over the years, and obesity is considered to be a chronic disease; therefore, such factors may have a considerable negative effect on future disease incidence. Our investigation pointed out that BMI contributed to multimorbidity, which is consistent with the finding of multimorbidity being highly associated with an increasing BMI and obesity [18,22,40,41]. A recent study also showed a dose-response relationship between the BMI and prevalence of multimorbidity [42]. In China, a study on 5493 Chinese adults aged 65 years or older found that a high level of obesity was associated with a high risk of multimorbidity [23]. A survey in Macau in 2016 [43] demonstrated that overweight and obese people accounted for 30.6% of the total population, with the highest incidence among individuals aged 45–59 years (38.5%). As obesity has become a common health problem in Macau, how to reduce multimorbidity in such individuals has become an important public health issue in society. 

In this study, we demonstrated the correlation between drinking and multimorbidity, that is, the participants who had quit drinking were likely to have multimorbidity; however, some studies showed no correlation between such variables [18,44]. Our results contradict those of Franken (2022), which emphasised that alcohol consumption is a protective factor against multimorbidity [42]. Another study determined that multimorbidity is highly prevalent among individuals (aged 50 years or older) who do not drink alcohol [45]. However, consistent with our findings, a study of 10,197 middle-aged and elderly Chinese individuals found that the former drinkers were likely to suffer from multimorbidity [13]. The inconsistent results may be because people with multimorbidity stop consuming alcohol after receiving a chronic-disease diagnosis. Thus, the temporality of the association should be investigated in longitudinal studies.

Our results also demonstrated that poor sleep quality was related to the prevalence of multimorbidity in older adults. Several studies reported the same findings [46,47,48]. Specifically, with the onset of ageing, melatonin levels decline, and circadian oscillations become less pronounced, making it harder for older adults to fall asleep and stay asleep compared with younger adults; hence, the quality of sleep of older adults declines gradually [49]. In addition, older adults are likely to be socially isolated, which can lead to sleep problems and poor sleep quality [50,51]. The appropriate amount of restful sleep is believed to be essential to people’s health and functioning [52,53]. Our study suggested that paying considerable attention to the sleep status of older adults and further investigating the mechanisms between sleep disorders and multimorbidity are necessary to reduce the prevalence of multimorbidity.

In our study, the most common disease combination was hypertension and hyperlipidaemia, followed by hypertension and diabetes. Hypertension, hyperlipidaemia and diabetes were the main chronic conditions among our study population, with a higher prevalence in the men than in the women, which is consistent with the findings in Macau [43] and Mainland China [54]. Previous studies showed a decreasing trend in hypertension in adults living in Macau, from 34.0% in 2012 [55] to 25.5% in 2016 [43]. However, our study demonstrated a continuing trend, that is, 19.4% in the men and 14.4% in the women. In the United States, the age-adjusted prevalence of hypertension in adults (aged ≥ 20 years) decreased from 1999 to 2016 [56]. This decreasing trend may be due to the increased use of antihypertensive medication among diagnosed individuals. Osteoporosis was the third most frequently reported chronic condition among the female participants, which accounted for 7.0% of the total sample. This result is consistent with the results of studies in Taiwan [57] and Mainland China [58,59]. Therefore, the increasing incidence of osteoporosis in the female population requires attention.

The participants were likely to have multimorbidity if they were older than 65 years, completed primary education or lower, were coupled and were retired/a housewife. Our results are consistent with those of a previous study that found that age, a low socioeconomic status and gender are important determinants of multimorbidity. As a literature review, the study included 39 studies conducted in 12 countries and involved more than 70 million patients [60]. As a special administrative region of China, Macau bears a heavy health burden caused by ageing. Age was identified as an important determinant of multimorbidity [60], and our results are consistent with those of previous studies in China [23,61] and Canada [25], which found that multimorbidity is common among elderly people. The analysis confirmed our finding that the higher the level of education, the lower the incidence of multimorbidity, which is consistent with research reports on New Zealand and Europe [14,16]. However, two studies conducted in Mainland China reported that multimorbidity increases in individuals with a low [2] or high education level [8]. However, data from the southern states of Brazil, which are highly developed in terms of education, showed an increase in the occurrence of multimorbidity among the residents [62]. People with a low education level may have fewer opportunities to access medical resources compared with those who are well educated. Moreover, the prevalence of multimorbidity is significantly higher in people who are socioeconomically disadvantaged compared with their counterparts, that is, in those with low income [5] and those who are unemployed [13,41] or retired [5]. A Brazilian study showed that unemployed adults are nearly 20% more likely to have multimorbidity than those who are employed [63]. We obtained similar results, probably because people with a low socioeconomic status are likely to engage in unhealthy behaviours, such as smoking, and are not likely to have access to preventive healthcare services.

Our study has several limitations. Firstly, though the hierarchical model seemed to account for the variables that may have reverse causality, the cross-sectional design did not allow us to establish a causal relationship between exposure and outcome. Secondly, we did not consider measures of common mental health disorders in our study. Finally, the cross-sectional design could not establish causality; thus, our findings may not be generalisable to populations with characteristics that differ from those of our research population. 

Despite such limitations, the findings of our community assessment can provide relatively reliable information about the health conditions of the Macau population. Our study is the first to investigate multimorbidity in a representative sample of Macau residents. Our use of a comprehensive lifestyle score to demonstrate the overall lifestyle status can provide local community stakeholders with quantitative evidence to recommend positive changes in lifestyle behaviours. The important implications of our study for individuals and society include the use of a simple and quantitative approach and education of people to change their lifestyle to avoid multimorbidity.

## 5. Conclusions

Our study highlights the heavy burden of multimorbidity among Chinese adults in Macau, which can help raise awareness among healthcare professionals and policymakers about the need to prevent and manage chronic diseases. The BMI, drinking, sleep quality and composite lifestyle score were significantly associated with multimorbidity, and the healthier the lifestyle, the lower the risk of multimorbidity. Our research findings can help guide the development of multimodal and efficient health promotion programmes that can target multiple lifestyle factors simultaneously to minimise the incidence of multimorbidity.

## Figures and Tables

**Table 1:** Construction and weighted score of lifestyle score.

Score	BMI	Total Physical Activity	Alcohol Consumption	Smoking Status	Sleep Quality
0 (poor)	≥25	no	≥8 dose/week for women or ≥15 dose/week for men	Current smokers	PSQI score > 5
1 (intermediate)	23–24.9	<150 min	no	Former smokers	PSQI score ≤ 5
2 (optimal)	<23	≥150 min	1–7 dose/week for women or 1–14 dose/week for men	Never	-
Total score			0–9		

**Table 2:** Basic and lifestyle characteristics stratified by multimorbidity status.

	Overall	Stratified by Multimorbidity Status		Effect Size ^a^	*p*-Value ^b^
		No	Yes		
**No. of participants**	1443	1294	149		
**Baseline characteristics**					
**Gender, n (%)**				0.01	0.755
Men	646 (44.8)	577 (44.6)	69 (46.3)		
Women	797 (55.2)	717 (55.4)	80 (53.7)		
**Age group, n (%)**				0.35	<0.001
Young Adult (18–44)	696 (48.2)	682 (52.7)	14 (9.4)		
Middle Age (45–64)	555 (38.5)	487 (37.6)	68 (45.6)		
Elderly (≥65)	192 (13.3)	125 (9.7)	67 (45.0)		
**Educational Level, n (%)**				0.25	<0.001
Primary or lower	280 (19.4)	210 (16.2)	70 (47.0)		
Secondary	723 (50.1)	658 (50.9)	65 (43.6)		
Tertiary or above	440 (30.5)	426 (32.9)	14 (9.4)		
**Marital status, n (%)**				0.11	<0.001
Single	540 (37.4)	508 (39.3)	32 (21.5)		
Couple	903 (62.6)	786 (60.7)	117 (78.5)		
**Employment status, n (%)**				0.29	<0.001
Employed	1042 (72.2)	978 (75.6)	64 (43.0)		
Unemployed	176 (12.2)	161 (12.4)	15 (10.1)		
Retired/Housewife	225 (15.6)	155 (12.0)	70 (47.0)		
**Monthly family income, n (%)**				0.03	0.778
$29,999 or less	718 (49.8)	642 (49.6)	76 (51.0)		
$30,000–$59,999	394 (27.3)	353 (27.3)	41 (27.5)		
$60,000 or more	108 (7.5)	100 (7.7)	8 (5.4)		
Unknown	223 (15.5)	199 (15.4)	24 (16.1)		
**Lifestyle characteristics**					
**BMI category, n (%)**				0.20	<0.001
Underweight/Normal weight (<23.0 kg/m^2^)	873 (60.5)	823 (63.6)	50 (33.6)		
Overweight (23–24.9 kg/m^2^)	283 (19.6)	244 (18.9)	39 (26.2)		
Obesity (≥25 kg/m^2^)	287 (19.9)	227 (17.5)	60 (40.3)		
**VPA Weekly, n (%)**				0.06	0.03
Not enough (<75 min)	1157 (80.2)	1027 (79.4)	130 (87.2)		
Enough (≥75 min)	286 (19.8)	267 (20.6)	19 (12.8)		
**MPA Weekly, n (%)**				0.02	0.43
Not enough (<150 min)	1141 (79.1)	1019 (78.7)	122 (81.9)		
Enough (≥150 min)	302 (20.9)	275 (21.3)	27 (18.1)		
**TOPA Weekly, n (%)**				0.06	0.03
Not enough (<150 min)	989 (68.5)	875 (67.6)	114 (76.5)		
Enough (≥150 min)	454 (31.5)	419 (32.4)	35 (23.5)		
**Drinking status, n (%)**				0.11	<0.001
Never	981 (68.0)	890 (68.8)	91 (61.1)		
Quitted	97 (6.7)	75 (5.8)	22 (14.8)		
Yes	365 (25.3)	329 (25.4)	36 (24.2)		
**Smoking, n (%)**				0.11	<0.001
Never	1200 (83.2)	1090 (84.2)	110 (73.8)		
Quitted	127 (8.8)	100 (7.7)	27 (18.1)		
Yes	116 (8.0)	104 (8.0)	12 (8.1)		
**Sleep quality, n (%)**				0.09	0.001
Optimal (PSQI score ≤ 5)	977 (67.7)	934 (69.1)	1337 (55.7)		
Poor/Insomnia (PSQI score > 5)	466 (32.3)	400 (30.9)	66 (44.3)		
**Lifestyle score, mean (SD)**	6.09 (1.56)	6.19 (1.52)	5.17 (1.57)	0.67	<0.001

VPA: Vigorous physical activity; MPA: moderate physical activity; TOPA weekly: weekly total physical activity; PSQI: Pittsburgh Sleep Quality Index; SD, standard deviation. ^a^ Cramer’s V for Chi-Square test of categorical variables and Cohen’s d for *t*-test of continuous variables. ^b^ Chi-Square test for categorical variables and *t*-test for continuous variables.

**Table 3:** Associations between lifestyle factors and lifestyle score and multimorbidity among Macau population.

Lifestyle Characteristics	Multimorbidity, Cases (%)	Model 1		Model 2	
		OR (95% CI)	*p*-Value	OR (95% CI)	*p*-Value
				**Model 2a**	
**BMI category**					
Underweight/Normal weight (<23.0 kg/m^2^)	50 (5.7)	Ref	-	Ref	-
Overweight (23–24.9 kg/m^2^)	39 (13.8)	2.63 (1.69, 4.09)	<0.001	1.95 (1.18, 3.20)	0.009
Obesity (≥25 kg/m^2^)	60 (20.9)	4.35 (2.91, 6.51)	<0.001	3.76 (2.38, 5.96)	<0.001
**VPA Weekly**					
Not enough (<75 min)	130 (11.2)	Ref	-	Ref	-
Enough (≥75 min)	19 (6.6)	0.56 (0.34, 0.93)	0.02	0.83 (0.44, 1.54)	0.55
**MPA Weekly**					
Not enough (<150 min)	122 (10.7)	Ref	-	Ref	-
Enough (≥150 min)	27 (8.9)	0.82 (0.53, 1.27)	0.37	1.00 (0.58, 1.74)	0.99
**Drinking status**					
Never	91 (9.3)	Ref	-	Ref	-
Quit	22 (22.7)	2.87 (1.70, 4.83)	<0.001	2.43 (1.26, 4.69)	0.008
Yes	36 (9.9)	1.07 (0.71, 1.61)	0.74	1.36 (0.81, 2.26)	0.24
**Smoking status**					
Never	110 (9.2)	Ref	-	Ref	-
Quit	27 (21.3)	2.68 (1.68, 4.27)	<0.001	1.16 (0.64, 2.09)	0.63
Yes	12 (10.3)	1.14 (0.61, 2.14)	0.68	0.74 (0.34, 1.62)	0.46
**Sleep quality**					
Optimal (PSQI score ≤ 5)	83 (8.5)	Ref	-	Ref	-
Poor/Insomnia (PSQI score > 5)	66 (14.2)	1.78 (1.26, 2.51)	0.001	2.25 (1.49, 3.40)	<0.001
				**Model 2b**	
**Lifestyle score**	-	0.64 (0.57, 0.72)	<0.001	0.67 (0.59, 0.77)	<0.001

VPA: Vigorous physical activity; MPA: moderate physical activity; PSQI: Pittsburgh Sleep Quality Index. Model 1 was a univariate logistic regression model; one regression model only included one lifestyle factor or lifestyle score as the independent variable; crude OR was calculated. Model 2a was a multivariable logistic regression model; BMI, VPA, MPA, drinking status, smoking status and insomnia were entered in one model for analysis, with adjustment of gender, age, employment status, education level, marital status and monthly family income. Model 2b was a multivariable logistic regression model; lifestyle score was entered in the model for analysis, with adjustment of gender, age, employment status, education level, marital status and monthly family income.

## Data Availability

Not applicable.

## Supplementary Materials

The following supporting information can be downloaded at: https://www.mdpi.com/article/10.3390/healthcare11131906/s1, Figure S1: Study flow diagram; Table S1: Sample estimation in each age group with reference to Macau Census data; Table S2: Associations between lifestyle factors and lifestyle score and multimorbidity among Macau population stratified by gender; Table S3: Associations between lifestyle factors and lifestyle score and multimorbidity among Macau population stratified by age; Table S4: Sensitivity analysis of associations between lifestyle factors and lifestyle score and multimorbidity among Macau population by excluding people with only one chronic disease; Table S5: Sensitivity analysis of associations between lifestyle factors and lifestyle score and multimorbidity among Macau population by excluding people who had no chronic disease; Table S6: Sensitivity analysis of associations between lifestyle factors and multimorbidity among Macau population by including total physical activity in the multivariable model.
