# Supplementary material for: The Multimorbidity and Lifestyle Correlates in Chinese Population Residing in Macau: Findings from a Community-Based Needs Assessment Study
Source: Healthcare (Basel). 2023 Jun 30;11(13):1906. doi: 10.3390/healthcare11131906 (PMC10341319; doi:10.3390/healthcare11131906)
Supplement: Supplementary file 1 [file healthcare-11-01906-s001.zip › healthcare-2425261-supplementary.pdf]

Supplementary Materials

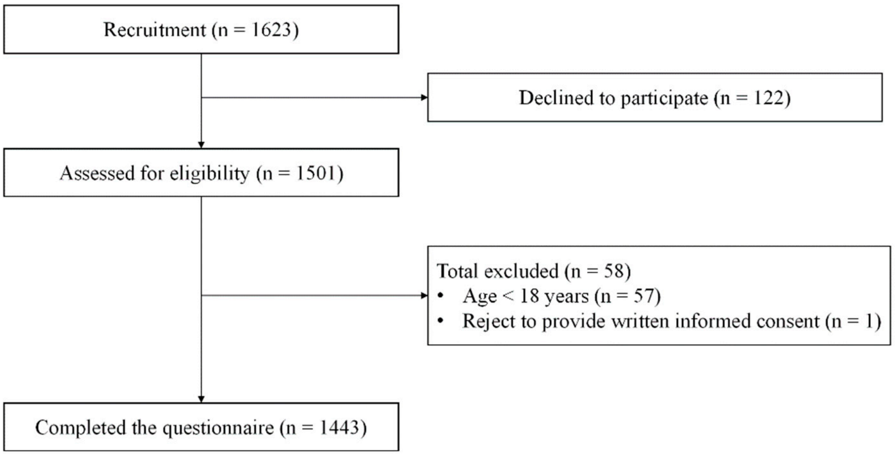

Figure S1. Study flow diagram.

Table S1. Sample estimation in each age group with reference to Macau Census data.

Age group	Macau Census data			Sample estimation		
	**Overall**	**Men**	**Women**	**Overall**	**Men**	**Women**
18-24	65849	36121	29728	129	71	58
25-34	135016	67321	67695	265	132	133
35-44	101537	48367	53170	200	95	105
45-54	104006	45528	58478	204	89	115
55-64	87583	42879	44704	172	84	88
>=65	59383	28167	31216	117	55	62
Total	553374	268383	284991	1087	526	561

Ref: Detailed Results of 2016 Population By-Census of Macau.

Table S2. Associations between lifestyle factors and lifestyle score and multimorbidity among Macau population stratified by gender.

Lifestyle characteristics	Men				
	Multimorbidity, cases (%)	Model 1		Model 2	
		OR (95% CI)	P-value	OR (95% CI)	P-value
				Model 2a	
**BMI category**					
Underweight/Normal weight (<23.0 kg/m2)	24 (6.7)	Ref	-	Ref	-
Overweight (23-24.9 kg/m2)	18 (11.5)	1.81 (0.95, 3.44)	0.07	1.55 (0.73, 3.29)	0.26
Obesity (≥25 kg/m2)	27 (20.3)	3.53 (1.96, 6.39)	< 0.001	3.52 (1.69, 7.36)	< 0.001
**VPA Weekly**					
Not enough (< 75 min)	59 (12.4)	Ref	-	Ref	-
Enough (>= 75 min)	10 (5.9)	0.45 (0.22, 0.89)	0.02	0.65 (0.26, 1.59)	0.34
**MPA Weekly**					
No enough (< 150 min)	54 (11.0)	Ref	-	Ref	-
Enough (>= 150 min)	15 (9.6)	0.85 (0.47, 1.55)	0.6	1.27 (0.58, 2.78)	0.55
**Drinking status**					
Never	27 (7.4)	Ref	-	Ref	-
Quitted	18 (26.9)	4.59 (2.35, 8.94)	< 0.001	2.80 (1.23, 6.38)	0.01
Yes	24 (11.2)	1.57 (0.88, 2.80)	0.13	1.53 (0.74, 3.19)	0.25
**Smoking status**					
Never	36 (7.8)	Ref	-	Ref	-
Quitted	23 (24.7)	3.87 (2.16, 6.92)	< 0.001	1.52 (0.74, 3.12)	0.25
Yes	10 (10.8)	1.42 (0.68, 2.97)	0.35	0.68 (0.27, 1.73)	0.42
**Insomnia (PSQI score >5)**					
Optimal <= 5	41 (8.6)	Ref	-	Ref	-
Poor > 5	28 (16.5)	2.09 (1.25, 3.51)	0.005	2.57 (1.33, 4.98)	0.005
				Model 2b	
**Lifestyle score**	-	0.65 (0.55, 0.76)	< 0.001	0.70 (0.58, 0.84)	< 0.001

Table S2. Associations between lifestyle factors and lifestyle score and multimorbidity among Macau population stratified by gender (Continued)

Lifestyle characteristics	Women				
	Multimorbidity, cases (%)	Model 1		Model 2	
		OR (95% CI)	P-value	OR (95% CI)	P-value
				Model 2a	
**BMI category**					
Underweight/Normal weight (<23.0 kg/m2)	26 (5.0)	Ref	-	Ref	-
Overweight (23-24.9 kg/m2)	21 (16.5)	3.73 (2.02, 6.89)	< 0.001	2.70 (1.37, 5.35)	0.004
Obesity (≥25 kg/m2)	33 (21.4)	5.14 (2.96, 8.92)	< 0.001	4.44 (2.38, 8.26)	< 0.001
**VPA Weekly**					
Not enough (< 75 min)	71 (10.4)	Ref	-	Ref	-
Enough (>= 75 min)	9 (7.7)	0.71 (0.35, 1.47)	0.36	1.17 (0.48, 2.86)	0.72
**MPA Weekly**					
Not enough (< 150 min)	68 (10.4)	Ref	-	Ref	-
Enough (>= 150 min)	12 (8.3)	0.77 (0.41, 1.47)	0.44	0.73 (0.32, 1.66)	0.46
**Drinking status**					
Never	64 (10.4)	Ref	-	Ref	-
Quitted	4 (13.3)	1.33 (0.45, 3.93)	0.61	2.46 (0.68, 8.85)	0.17
Yes	12 (8.0)	0.75 (0.39, 1.43)	0.38	1.11 (0.51, 2.42)	0.78
**Smoking status**					
Never	74 (10.0)	Ref	-	Ref	-
Quitted	4 (11.8)	1.20 (0.41, 3.50)	0.74	0.58 (0.17, 2.00)	0.39
Yes	2 (8.7)	0.86 (0.20, 3.73)	0.84	1.41 (0.27, 7.36)	0.68
**Insomnia (PSQI score >5)**					
Optimal <= 5	42 (8.4)	Ref	-	Ref	-
Poor > 5	38 (12.8)	1.61 (1.01, 2.56)	0.04	2.00 (1.17, 3.45)	0.01
				Model 2b	
**Lifestyle score**	-	0.61 (0.51, 0.73)	< 0.001	0.63 (0.52, 0.78)	< 0.001

VPA: Vigorous physical activity; MPA: moderate physical activity; PSQI: Pittsburg Sleep Quality Index.

Model 1 was univariate logistic regression model, one regression model only included one lifestyle factor or lifestyle score as the independent variable; crude OR was calculated; Model 2a was multivariable logistic regression model, BMI, VPA, MPA, drinking status, smoking status, insomnia were put in one model for analysis, with adjustment of gender, age, employment status, education level, marital status, and monthly family income. Model 2b was multivariable logistic regression model, lifestyle score was put in the model for analysis, with adjustment of gender, age, employment status, education level, marital status, and monthly family income.

Table S3. Associations between lifestyle factors and lifestyle score and multimorbidity among Macau population stratified by age.

Lifestyle characteristics	Multimorbidity, cases (%)	age < 45 y			
		Model 1		Model 2	
		OR (95% CI)	P-value	OR (95% CI)	P-value
				Model 2a	
**BMI category**					
Underweight/Normal weight (<23.0 kg/m2)	6 (1.2)	Ref	-	Ref	-
Overweight (23-24.9 kg/m2)	2 (2.1)	1.70 (0.34, 8.56)	0.52	1.30 (0.20, 8.26)	0.78
Obesity (≥25 kg/m2)	6 (5.6)	4.75 (1.50, 15.04)	0.008	6.88 (1.68, 28.12)	0.007
**VPA Weekly**					
Not enough (< 75 min)	14 (2.7)	Ref	-	Ref	-
Enough (>= 75 min)	0 (0.0)	-[c]	-[c]	-[c]	-[c]
**MPA Weekly**					
Not enough (< 150 min)	14 (2.6)	Ref	-	Ref	-
Enough (>= 150 min)	0 (0.0)	-[c]	-[c]	-[c]	-[c]
**Drinking status**					
Never	9 (2.0)	Ref	-	Ref	-
Quitted	1 (3.0)	1.55 (0.19, 12.61)	0.68	1.49 (0.12, 18.98)	0.76
Yes	4 (1.9)	0.97 (0.30, 3.19)	0.96	0.80 (0.18, 3.62)	0.77
**Smoking status**					
Never	11 (1.8)	Ref	-	Ref	-
Quitted	2 (6.9)	4.10 (0.87, 19.42)	0.08	2.65 (0.29, 23.91)	0.38
Yes	1 (2.1)	1.20 (0.15, 9.53)	0.86	0.79 (0.05, 11.98)	0.86
**Insomnia (PSQI score >5)**					
Optimal <= 5	6 (1.3)	Ref	-	Ref	-
Poor > 5	8 (3.5)	2.74 (0.94, 8.01)	0.06	2.48 (0.68, 9.06)	0.17
				Model 2b	
**Lifestyle score**	-	0.45 (0.31, 0.64)	< 0.001	0.49 (0.32, 0.74)	< 0.001

Table S3. Associations between lifestyle factors and lifestyle score and multimorbidity among Macau population stratified by age (Continued)

Lifestyle characteristics	age >= 45 y				
	Multimorbidity, cases (%)	Model 1		Model 2	
		OR (95% CI)	P-value	OR (95% CI)	P-value
				Model 2a	
**BMI category**					
Underweight/Normal weight (<23.0 kg/m2)	44 (11.5)	Ref	-	Ref	-
Overweight (23-24.9 kg/m2)	37 (19.9)	1.91 (1.18, 3.08)	0.008	2.20 (1.31, 3.69)	0.003
Obesity (≥25 kg/m2)	54 (30.2)	3.32 (2.12, 5.19)	< 0.001	3.48 (2.13, 5.68)	< 0.001
**VPA Weekly**					
Not enough (< 75 min)	116 (18.0)	Ref	-	Ref	-
Enough (>= 75 min)	19 (18.4)	1.03 (0.60, 1.76)	0.92	1.10 (0.57, 2.13)	0.77
**MPA Weekly**					
Not enough (< 150 min)	108 (17.8)	Ref	-	Ref	-
Enough (>= 150 min)	27 (19.3)	1.10 (0.69, 1.76)	0.68	1.12 (0.62, 2.01)	0.71
**Drinking status**					
Never	82 (15.6)	Ref	-	Ref	-
Quitted	21 (32.8)	2.64 (1.49, 4.69)	< 0.001	2.43 (1.22, 4.83)	0.01
Yes	32 (20.4)	1.39 (0.88, 2.18)	0.16	1.42 (0.82, 2.46)	0.22
**Smoking status**					
Never	99 (17.1)	Ref	-	Ref	-
Quitted	25 (25.5)	1.66 (1.01, 2.75)	0.05	1.12 (0.61, 2.06)	0.71
Yes	11 (15.9)	0.92 (0.47, 1.82)	0.81	0.73 (0.32, 1.67)	0.46
**Insomnia (PSQI score >5)**					
Optimal <= 5	77 (15.0)	Ref	-	Ref	-
Poor > 5	58 (24.7)	1.85 (1.26, 2.72)	0.002	2.04 (1.32, 3.17)	0.001
				Model 2b	
**Lifestyle score**	-	0.76 (0.66, 0.86)	< 0.001	0.73 (0.63, 0.84)	< 0.001

VPA: Vigorous physical activity; MPA: moderate physical activity; PSQI: Pittsburg Sleep Quality Index.

Model 1 was univariate logistic regression model, one regression model only included one lifestyle factor or lifestyle score as the independent variable; crude OR was calculated.

Model 2a was multivariable logistic regression model, BMI, VPA, MPA, drinking status, smoking status, insomnia were put in one model for analysis, with adjustment of gender, age, employment status, education level, marital status, and monthly family income.

Model 2b was multivariable logistic regression model, lifestyle score was put in the model for analysis, with adjustment of gender, age, employment status, education level, marital status, and monthly family income.

[c] the result was not reported due to the absence of multimorbidity case.

Table S4. Sensitivity analysis of associations between lifestyle factors and lifestyle score and multimorbidity among Macau population by excluding people with only one chronic disease.

Lifestyle characteristics	Multimorbidity, cases (%)	Model 1		Model 2	
		OR (95% CI)	P-value	OR (95% CI)	P-value
				Model 2a	
**BMI category**					
Underweight/Normal weight (<23.0 kg/m2)	50 (6.9)	Ref	-	Ref	-
Overweight (23-24.9 kg/m2)	39 (18.1)	3.01 (1.92, 4.72)	< 0.001	2.39 (1.38, 4.14)	0.002
Obesity (≥25 kg/m2)	60 (28.2)	5.33 (3.52, 8.06)	< 0.001	5.08 (3.04, 8.48)	< 0.001
**VPA Weekly**					
Not enough (< 75 min)	130 (14.3)	Ref	-	Ref	-
Enough (>= 75 min)	19 (7.8)	0.51 (0.31, 0.84)	0.008	0.71 (0.37, 1.38)	0.31
**MPA Weekly**					
Not enough (< 150 min)	122 (13.5)	Ref	-	Ref	-
Enough (>= 150 min)	27 (10.8)	0.77 (0.50, 1.21)	0.26	0.95 (0.52, 1.74)	0.87
**Drinking status**					
Never	91 (11.6)	Ref	-	Ref	-
Quitted	22 (31.4)	3.50 (2.02, 6.06)	< 0.001	2.39 (1.12, 5.11)	0.02
Yes	36 (11.9)	1.03 (0.68, 1.56)	0.88	1.01 (0.57, 1.76)	0.98
**Smoking status**					
Never	110 (11.2)	Ref	-	Ref	-
Quitted	27 (29.3)	3.28 (2.01, 5.36)	< 0.001	0.96 (0.48, 1.91)	0.9
Yes	12 (14.0)	1.28 (0.67, 2.43)	0.45	0.82 (0.34, 1.94)	0.65
**Insomnia (PSQI score >5)**					

Optimal <= 5	83 (10.8)	Ref	-	Ref	-
Poor > 5	66 (17.1)	1.71 (1.21, 2.42)	0.003	2.26 (1.44, 3.57)	< 0.001
				**Model 2b**	
**Lifestyle score**	**-**	0.60 (0.53, 0.68)	< 0.001	0.64 (0.55, 0.74)	< 0.001

VPA: Vigorous physical activity; MPA: moderate physical activity; PSQI: Pittsburg Sleep Quality Index.

Model 1 was univariate logistic regression model, one regression model only included one lifestyle factor or lifestyle score as the independent variable; crude OR was calculated.

Model 2a was multivariable logistic regression model, BMI, VPA, MPA, drinking status, smoking status, insomnia were put in one model for analysis, with adjustment of gender, age, employment status, education level, marital status, and monthly family income.

Model 2b was multivariable logistic regression model, lifestyle score was put in the model for analysis, with adjustment of gender, age, employment status, education level, marital status, and monthly family income.

Table S5. Sensitivity analysis of associations between lifestyle factors and lifestyle score and multimorbidity among Macau population by excluding people who had no chronic disease.

Lifestyle characteristics	Multimorbidity, cases (%)	Model 1		Model 2	
		OR (95% CI)	P-value	OR (95% CI)	P-value
				Model 2a	
**BMI category**					
Underweight/Normal weight (<23.0 kg/m2)	50 (25.8)	Ref	-	Ref	-
Overweight (23-24.9 kg/m2)	39 (36.4)	1.65 (0.99, 2.75)	0.05	1.43 (0.81, 2.52)	0.22
Obesity (≥25 kg/m2)	60 (44.8)	2.34 (1.46, 3.73)	< 0.001	2.12 (1.26, 3.58)	0.005
**VPA Weekly**					
Not enough (< 75 min)	130 (34.7)	Ref	-	Ref	-
Enough (>= 75 min)	19 (31.7)	0.87 (0.49, 1.57)	0.65	0.93 (0.45, 1.90)	0.83
**MPA Weekly**					
Not enough (< 150 min)	122 (34.2)	Ref	-	Ref	-
Enough (>= 150 min)	27 (34.6)	1.02 (0.61, 1.71)	0.94	1.03 (0.55, 1.92)	0.93
**Drinking status**					
Never	91 (31.7)	Ref	-	Ref	-
Quitted	22 (44.9)	1.75 (0.95, 3.25)	0.07	2.56 (1.22, 5.39)	0.01
Yes	36 (36.4)	1.23 (0.76, 1.99)	0.4	1.87 (1.02, 3.42)	0.04
**Smoking status**					
Never	110 (33.2)	Ref	-	Ref	-
Quitted	27 (43.5)	1.55 (0.89, 2.69)	0.12	1.30 (0.67, 2.54)	0.44
Yes	12 (28.6)	0.80 (0.40, 1.63)	0.54	0.63 (0.26, 1.48)	0.29
**Insomnia (PSQI score >5)**					

Optimal <= 5	83 (28.7)	Ref	-	Ref	-
Poor > 5	66 (45.2)	2.05 (1.35, 3.10)	< 0.001	2.46 (1.53, 3.95)	< 0.001
				**Model 2b**	
**Lifestyle score**	**-**	0.77 (0.68, 0.88)	< 0.001	0.76 (0.66, 0.88)	< 0.001

VPA: Vigorous physical activity; MPA: moderate physical activity; PSQI: Pittsburg Sleep Quality Index.
Model 1 was univariate logistic regression model, one regression model only included one lifestyle factor or lifestyle score as the independent variable; crude OR was calculated.
Model 2a was multivariable logistic regression model, BMI, VPA, MPA, drinking status, smoking status, insomnia were put in one model for analysis, with adjustment of gender, age, employment status, education level, marital status, and monthly family income.
Model 2b was multivariable logistic regression model, lifestyle score was put in the model for analysis, with adjustment of gender, age, employment status, education level, marital status, and monthly family income.

Table S6. Sensitivity analysis of associations between lifestyle factors and multimorbidity among Macau population by including total physical activity in the multivariable model.

Lifestyle characteristics	OR (95% CI)	P-value
**BMI category**		
Underweight/Normal weight (<23.0 kg/m2)	Ref	-
Overweight (23-24.9 kg/m2)	1.95 (1.18, 3.20)	0.009
Obesity (≥25 kg/m2)	3.80 (2.40, 6.02)	< 0.001
**TOPA Weekly**		
Not enough (< 150 min)	Ref	-
Enough (>= 150 min)	0.82 (0.51, 1.31)	0.4
**Drinking status**		
Never	Ref	-
Quitted	2.45 (1.27, 4.72)	0.008
Yes	1.35 (0.81, 2.25)	0.26
**Smoking status**		
Never	Ref	-
Quitted	1.18 (0.65, 2.12)	0.59
Yes	0.75 (0.35, 1.64)	0.48
**Insomnia (PSQI score >5)**		
Optimal <= 5	Ref	-
Poor > 5	2.25 (1.49, 3.40)	< 0.001

VPA: Vigorous physical activity; TOPA weekly: weekly total physical activity; PSQI: Pittsburg Sleep Quality Index.

Multivariable logistic regression model, BMI, VPA, MPA, drinking status, smoking status, insomnia were put in one model for analysis, with adjustment of gender, age, employment status, education level, marital status, and monthly family income.
